# Monomolecular tetrahelix of polyguanine with a strictly defined folding pattern

**DOI:** 10.1038/s41598-018-28572-x

**Published:** 2018-07-04

**Authors:** Besik Kankia

**Affiliations:** Department of Chemistry and Biochemistry, The Ohio State University, Columbus, OH 43210 USA

## Abstract

The G_3_TG_3_TG_3_TG_3_ (G3T) sequence folds into a monomolecular quadruplex with all-parallel G_3_ segments connected to each other by chain-reversal loops. The homopolymer consisting of *n* number of G3T domains directly conjugated to each other folds into an uninterrupted and unusually stable polymer, tetrahelical monomolecular DNA (tmDNA). It was demonstrated that the tmDNA architecture has strong potential in nanotechnologies as highly programmable building material, high affinity coupler and the driving force for endergonic reactions. Here, we explore capability of analogous DNA sequences (i.e., monomolecular quadruplexes with G_2_ or G_4_ segments) to construct tmDNA architecture. The study demonstrates that tmDNA can have only one building pattern based on a quadruplex domain with three G-tetrads and single-nucleotide loops, G3N (N = G, A, C and T); all other domains demonstrate antiparallel topologies unsuitable for tmDNA. The present study also suggests that polyguanine is capable of tmDNA formation with strictly defined building pattern; G_3_ segments connected to each other by chain-reversal G-loops. These findings can have significant impact on (i) DNA nanotechnologies; (ii) structure prediction of G-rich sequences of genome; and (iii) modeling of abiogenesis.

## Introduction

**Figure 1:**
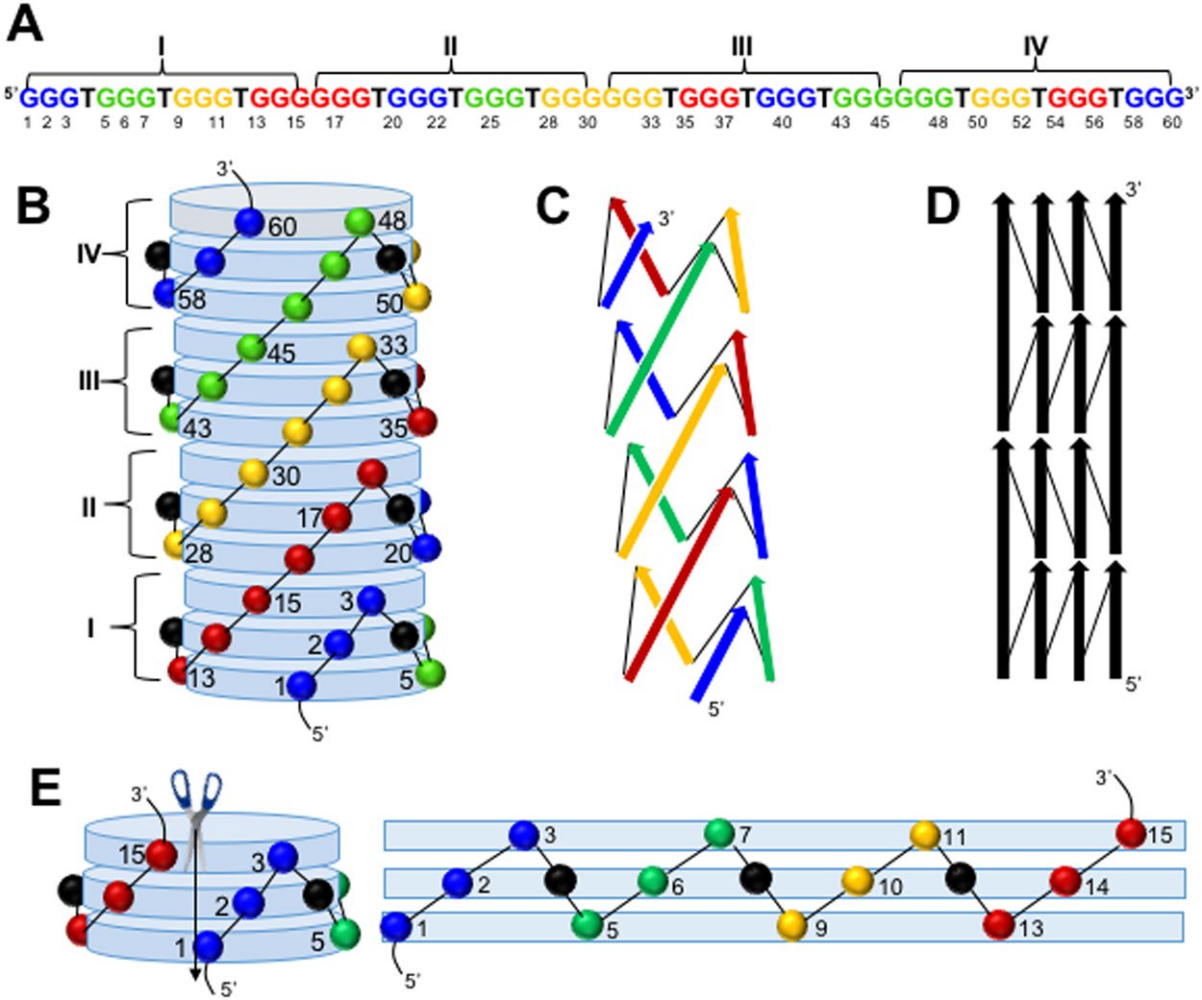
Model of G3T-tmDNA. (**A**) Sequence of (G3T)_4_. The Arabic and Roman numerals correspond to nucleotide and G3T domains positions. Color code helps to identify helices. For instance, the red helix is made of 13–18, 35–37, and 54–56 guanines with two interruptions. (**B**) Three-dimensional model of tmDNA viewed as a cylinder formed by stacked G-quartets (disks). The colored spheres represent sugars of guanines involved in G-quartets. The black spheres represent sugars of unstructured thymidines forming the propeller loops (thymines are omitted for clarity); (**C** and **D**) simplified 3D and 2D schemes of tmDNA. The arrows represent GGG segments and thin lines correspond to T-loops. (**E**) The first G3T domain of (G3T)_4_ in the unfolded state.

Recently, we have described a tetrahelical monomolecular DNA (tmDNA) that employs G_3_TG_3_TG_3_TG_3_ quadruplex as a structural domain (Fig. 1)^1^. The quadruplex domain is folded with all-parallel G_3_ segments connected to each other by chain-reversal or propeller loops (Fig. 1E)^2,3^. Hereafter, a quadruplex domain is called a DNA sequence with four G-tracts of equal length connected by three loop-segments of equal length. For instance, four G_3_-segments connected by three T-loops, shown above, is abbreviated to G3T, and G3T-tmDNA stands for the polymer built by G3T domain (Fig. 1). Similarly, G_4_T_2_G_4_T_2_G_4_T_2_G_4_ is abbreviated to G4T2 with hypothetical polymer G4T2-tmDNA; or G_15_ can be considered as four G_3_-segments connected by three G-loops and abbreviated to G3G, and tmDNA formed by a polyG can be designated as G3G-tmDNA.

G3T-tmDNA is visualized as a homopolymer consisting of *n* number of G3T domains, (G3T)_*n*_^1^. The terminal G_3_-segments of adjacent G3T domains are directly attached to each other forming G_6_-segments. As a result, (G3T)_*n*_ contains 2 × (*n* + 1) G_3_-segments and (*n* − 1) G_6_-segments (Fig. 1A). Each G3T domain of the architecture is formed by zigzagging of G_3_-segments and T-loops (Fig. 1E) while G_6_ segment is sheared by adjacent domains and serves as a bridge between them (Fig. 1B–D). The G_6_-segments are responsible for vertical growth of the structure while G_3_-segments and T-loops move DNA strand horizontally. In contrast, movement of a strand in DNA double helix is always vertical. The tmDNA represents the only nucleic acid structure, which is not based on base-pare complementarity and is capable of forming an uninterrupted polymer with specifically defined building pattern. The tmDNA has strong potential in DNA nanotechnology as a building material capable of producing monomolecular nanostructures^1,4^, high affinity coupler^5^ and driving force for endergonic reactions^6^.

**Figure 2:**
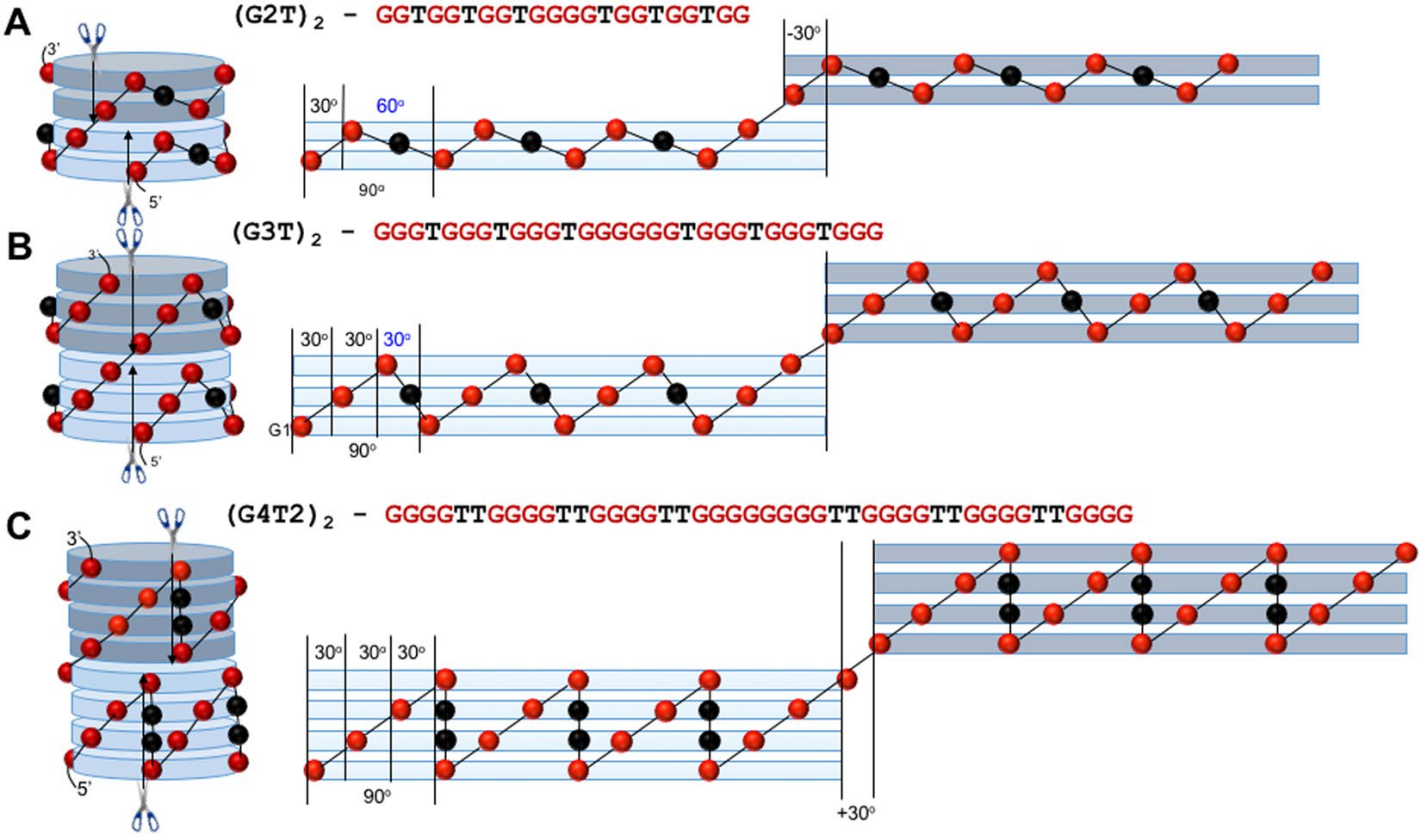
Potential architectures based on two (**A**), three (**B**) and four (**C**) G-tetrads. For clarity, only two domains are shown. Each domain is dissected at the 5′-end and unfolded: top domains clockwise and bottom domains counter clockwise. Angle of rotation between all adjacent guanines, τ(G-G), is equal to 30° in all models (lit), i.e., τ(G1-G2) in all three models; or τ(G3-G4), τ(G21-G22) and τ(G22-G23) in G4T2. The angel between guanines linked through T-loops is predetermined from 4-fold rotational symmetry of G-tetrads; τ(G-T-G) = 90° − (*n* − 1) × 30°, where *n* is number of Gs in G-tracts (i.e., 4 in G4T2). The τ(G-T-G) angles are shown in blue. They determine the helicity of the architecture in terms of domains (in terms of G-tetrads all models are the right-handed helixes with 12 G-tetrads per turn). For instance, G3T has no helicity; domains are stacked on top of each without rotation. However, (G2T)_n_ and (G4T2)_n_ are left-handed and right-handed helices, respectively, with 12 domains per turn.

Since cations are chelated between G-tetrads, thermal stability and folding topology of quadruplexes strongly depends on cation size^7^. Both monovalent and divalent cations with radii between ~100 pm (Na^+^ or Ca^2+^) and ~140 pm (K^+^ or Sr^2+^) are capable of quadruplex formation, while cations out of this interval (i.e., Li^+^ or Mg^2+^ with 70 pm and Cs^+^ with 167 pm) don’t fold quadruplexes. Another important factor determining stability and topology of the monomolecular quadruplexes is loop-length^8–11^. For instance, in the presence of K^+^ ions (the most favorable monovalent cation for quadruplex formation), G3T adopts homogeneous structure with all-parallel G_3_ segments and demonstrates high thermal stability. G3T version with elongated loops, G3T2, shows a structural polymorphism (mainly parallel quadruplex with some antiparallel topologies) and 25 °C drop in the stability^8,9,12^. The same sequence in the presence of Na^+^ ions, Na^+^-G3T2, demonstrates increased amount of the antiparallel topology and further drop in stability by 36 °C^8,9,12^. Further elongation of loop length (i.e., G3T3) has even stronger destabilizing effects^8,9^. Thus, both loop elongation and replacing K^+^ by less favorable cations (i.e., Na^+^) strongly destabilize G3T quadruplex and jeopardizes its structural homogeneity. The homogenous structure of the quadruplex domain with all-parallel topology is essential for tmDNA, since any antiparallel G_3_-segment with lateral or diagonal loops will inhibit stacking between quadruplex domains and ruin continuous fold of the architecture (see Fig. S1). Thus, G3T seems to be only quadruplex domain suitable for tmDNA among domains with three G-tetrads. The questions then arise: (i) does G3T-tmDNA fold in the presence of other alkali and alkaline-earth cations? (ii) Does tmDNA fold with other building patterns, i.e., quadruplex domains with two or four G-tetrads, G2T-tmDNA or G4T2-tmDNA (see Fig. 2)?

To address these questions, we employed UV thermal unfolding and circular dichroism (CD) and performed systematic study of G_2_, G_3_ and G_4_-based domains (i.e, G2T or G3T) and their corresponding dimers (i.e., (G2T)_2_ or (G3T)_2_). While the domains (four G-tracts of equal length connected by three loop-segment) were studied earlier (reviewed in Discussion), studies on the dimers, which favor tmDNA formation^1^, are missing. UV unfolding is very simple and accurate tool to monitor quadruplrx melting without any modifications of oligonucleotides^7,13^. In addition, UV melting is a bulk assay monitoring averaged value of molecular assemblies and therefore free from problems of single-molecular techniques (i.e., random behavior of molecules) and allows wide variation of experimental conditions (i.e., here we employ all alkali and alkaline-earth metal ions). Also, UV melting experiments can be easily performed on long constructs (i.e., quadruplex dimers), which is problematic for NMR studies. CD spectroscopy represents an accurate and straightforward assay to characterize overall topology of the quadruplexes. It is particularly useful to see whether all-parallel fold of K^+^-G3T experiences rearrangement into antiparallel topology (one of the main indicators of distorting tmDNA folding pattern) upon cation exchange or sequence modifications.

The present work demonstrates that tmDNA can have only one building pattern based on a quadruplex domain with three G-tetrads and single-nucleotide loops, G3N (N = G, A, C and T); all other domains are significantly destabilized and demonstrate antiparallel topologies unsuitable for tmDNA. The G3N-tmDNA demonstrates several unique properties, which is useful in DNA nanotechnologies and could play central role during abiogenesis.

## Results

### CD spectroscopy

CD is a useful technique for estimating folding topology of DNA quadruplexes containing regular G-segments connected with T-loops^14^. By comparative analysis of CD spectra and structural data of quadruplexes the following spectral characteristics have been observed: antiparallel quadruplexes demonstrate positive peaks at ∼245 nm and ∼295 nm and a negative peak at ∼265 nm, while parallel quadruplexes show a strong positive band at ∼265 nm and a negative peak of lesser intensity at ∼240 nm^15–17^. Characteristics of parallel quadruplexes also include a minor positive peak at ∼305 nm^15,18–20^. Although CD spectrometry cannot determine structural details of the quadruplexes, it can detect any alteration in all-parallel topology of G3T due to sequence modification.

**Figure 3:**
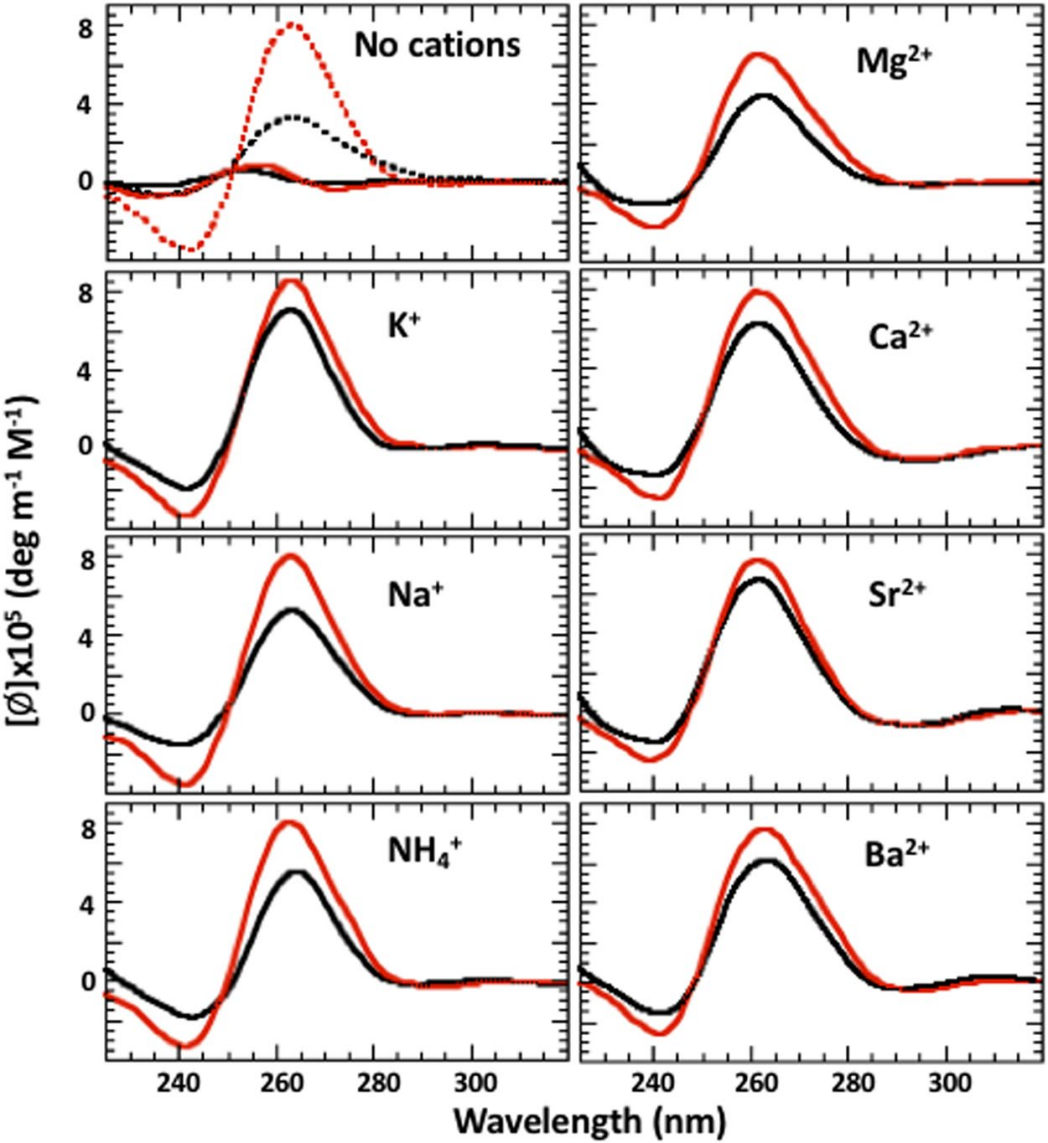
CD spectra of G3T (black) and (G3T)_2_ (red) in the presence of 25 mM monovalent and 5 mM divalent cations at 50 °C. The dashed curves correspond to the samples without added cations at 20 °C.

Structural properties of many monomolecular quadruplexes strongly depend on the procedure of sample preparation, especially the thermal steps. For proper comparison, all CD samples were treated similarly (see Materials and Methods). Figure 3 shows CD spectra of G3T and (G3T)_2_ in the presence of different cations (25 mM Me^+^ and 5 mM Me^2+^) at 50 °C. All spectra are consistent with an all-parallel topology. Thus, cation type and size does not have any effect on the topology. In the absence of cations (top left panel, Fig. 3), CD does not show any measurable signals at 50 °C (solid curves), however at 20 °C (dashed curves) it demonstrates all-parallel fold for both G3T and (G3T)_2_. This demonstrates that at room temperatures both G3T and (G3T)_2_ are capable of quadruplex formation even in the absence of added cations. In addition, CD profiles of the quadruplexe aren’t affected by the sample preparation (Fig. S2).

**Figure 4:**
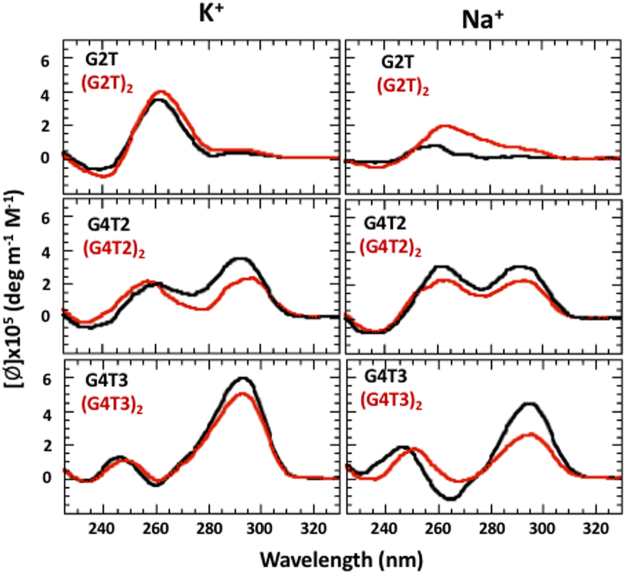
CD spectra of G_2_- and G_4_-containing quadruplexes in the presence of 50 mM KCl and NaCl ions at 20 °C. The black curves correspond to the domains or monomers and red curves correspond to the dimers.

Figure 4 shows CD spectra of G_2_- and G_4_-containing quadruplexes in the presence of 50 mM K^+^ and Na^+^ ions at 20 °C. G2T and (G2T)_2_, demonstrate similar profiles in the presence of K^+^ ions corresponding mainly parallel topology. However, positive peak around 295 nm indicates the presence of some anti-parallel fold. In the presence of Na^+^ ions, these sequences don’t form any stable structures. The G4T2 and (G4T2)_2_ sequences in Na^+^ demonstrate two positive peaks of similar amplitude at ~265 nm and ~295 nm suggesting both parallel and antiparallel topologies. In the presence of K^+^ ions both sequences show similar profiles with increased amount of antiparallel fold. CD profiles of G4T3 and the dimer demonstrates mostly antiparallel topology for both cations (lower panels, Fig. 4).

### UV melting curves of G3T and (G3T)2 in the presence of different cations

**Figure 5:**
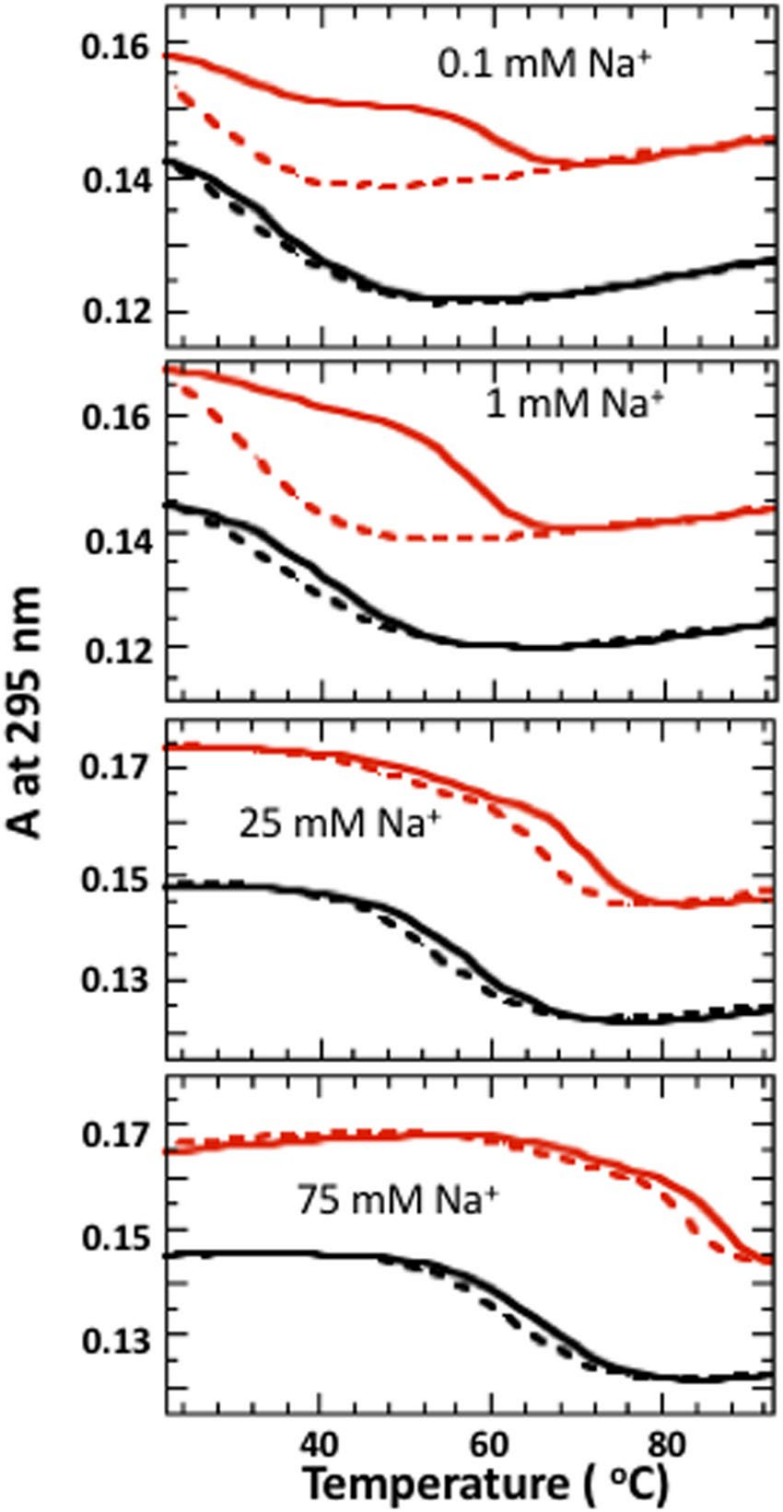
UV unfolding (solid) and refolding (dashed) curves of G3T (black) and (G3T)_2_ (red) in the presence of NaCl.

Temperature-dependent UV experiments of DNA quadruplexes are accompanied by a decrease in absorbance at 295 nm^7,13^. G3T demonstrates two-state transitions at all Na^+^ concentrations tested here (Fig. 5). However, (G3T)_2_ shows large hysteresis at lower cation concentrations. In the presence of 0.1 mM Na^+^ ions, unfolding curve of (G3T)_2_ shows biphasic transition. The first transition, at ∼30 °C, can be attributed to partially folded dimer due to insufficient amount of Na^+^ (i.e., (G3T)_2_ oligonucleotide with only one folded quadruplex). Indeed, at 1 mM Na^+^ first transition disappears while amplitude of the second transition increases (Fig. 5). With further increase of Na^+^ concentration hysteresis gradually decreases and at 75 mM both molecules, G3T and (G3T)_2_, demonstrate equilibrium transitions at 65 °C and 85 °C, respectively.

**Figure 6:**
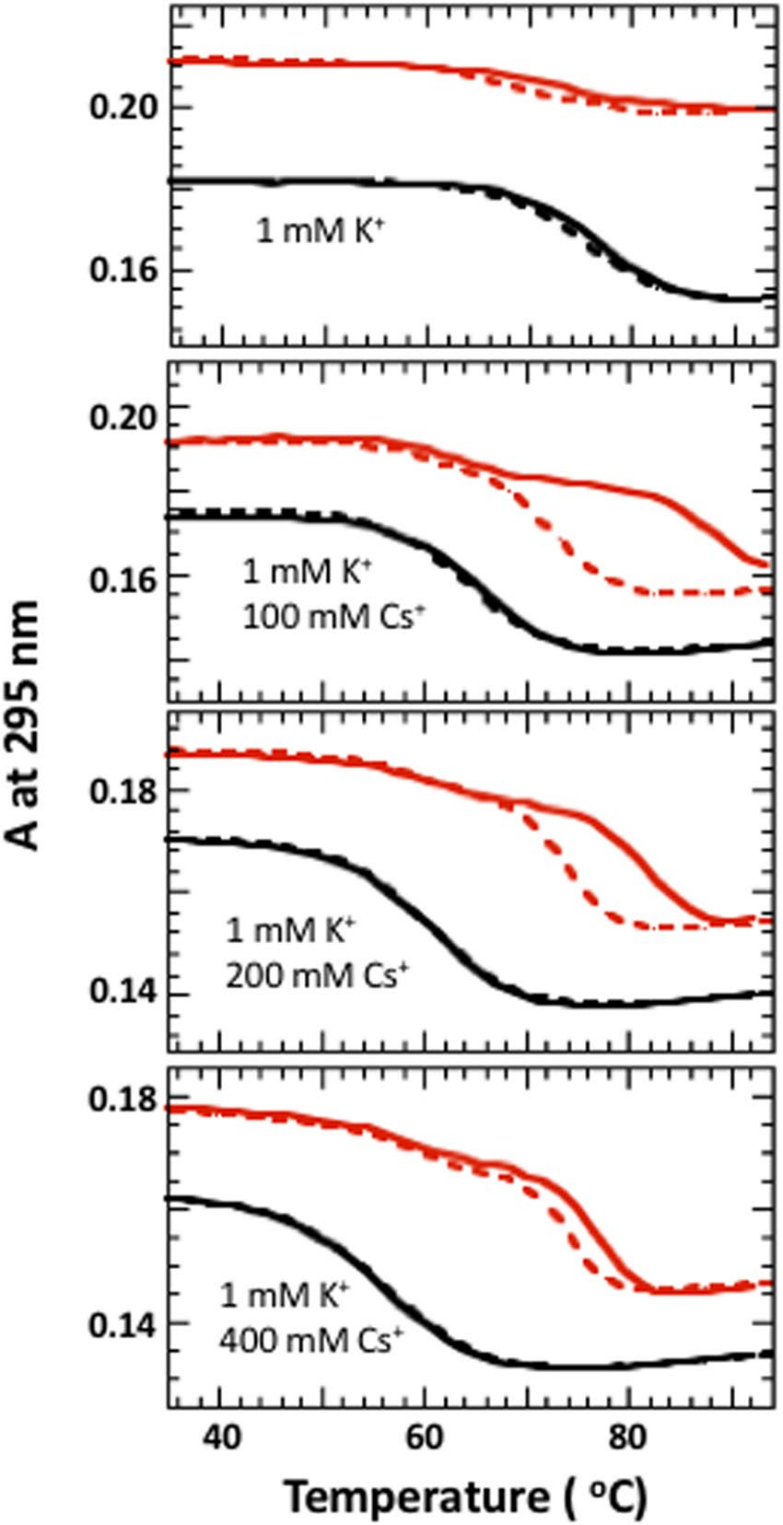
UV unfolding (solid) and refolding (dashed) curves of G3T (black) and (G3T)_2_ (red) in the presence of 1 mM KCl and different amounts of CsCl.

Figure 6 shows unfolding experiments of G3T and (G3T)_2_ formed by 1 mM K ions in the presence of varying amounts of CsCl. Before adding CsCl, (G3T)_2_ melts above the boiling point, however, adding 100 mM Cs^+^ brings it within measurable temperature interval. At 200 mM CsCl the hysteresis reduces and at 400 mM almost completely disappears.

**Figure 7:**
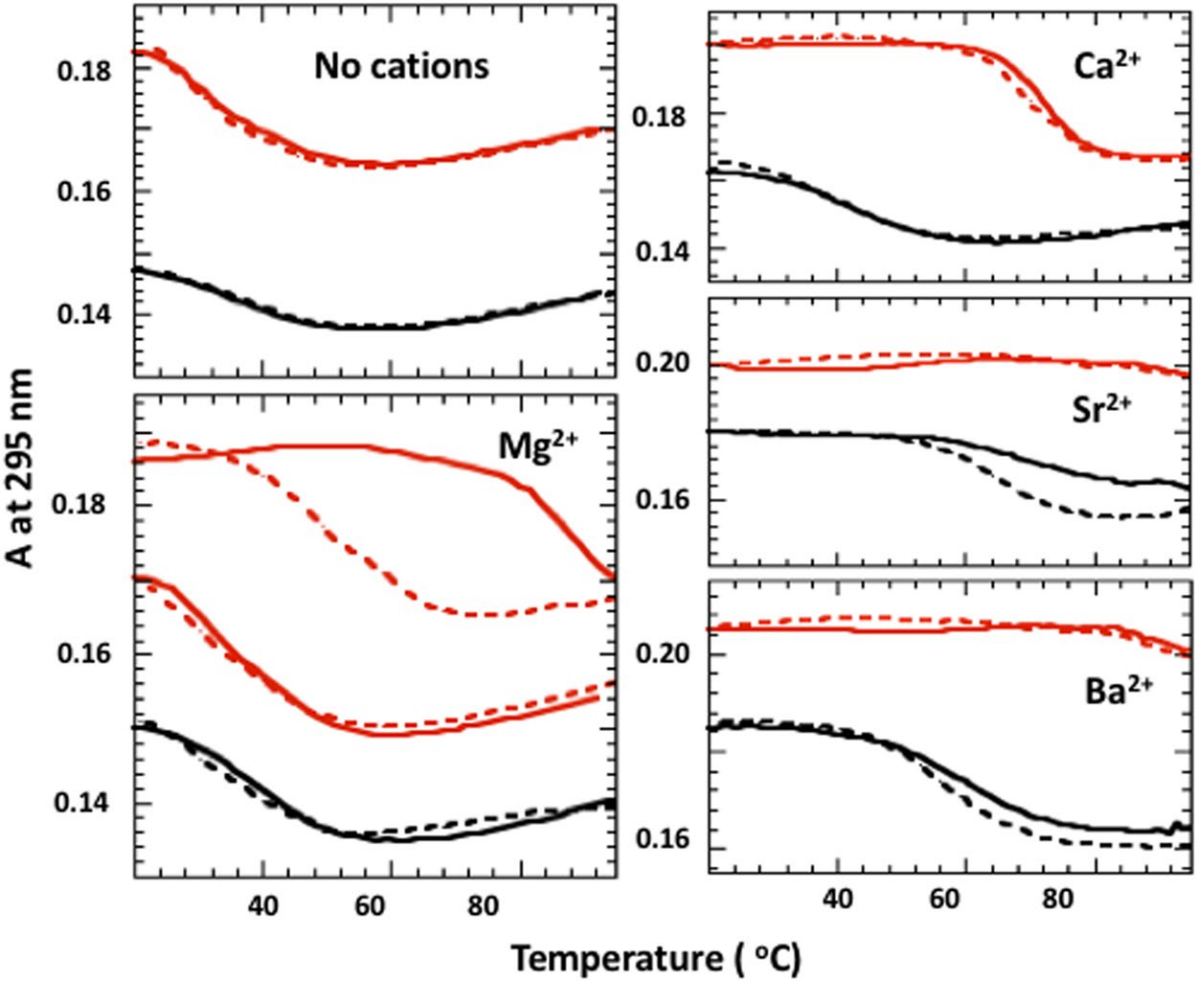
UV unfolding (solid) and refolding (dashed) curves of (G3T)_2_ (red) and G3T (black) in the presence of 50 µM divalent cations. Panel with MgCl_2_ show the melting experiment of (G3T)_2_ after adding 100 mM CsCl (middle red curves).

Figure 7 demonstrates melting experiments of G3T and (G3T)_2_ in the absence and presence of 50 µM alkaline-earth cations. Ca^2+^ ions demonstrate the least stabilization effect and allows to record entire transitions for both quadruplexes, which are fully reversible; G3T and (G3T)_2_ unfold at 40 °C and 70 °C, respectively. As expected, Sr^2+^ and Ba^2+^ show stronger stabilization effects, which did not allow to record unfolding of (G3T)_2_ (Fig. 7). Thus, all curves correspond to equilibrium transitions besides Sr^2+^-G3T and Mg^2+^-(G3T)_2_; the former is due G3T dimerization through end-to-end stacking^14^, while latter reminds hysteretic behavior of K^+^-(G3T)_2_ and should be induced by domain-domain interaction within the dimer^4^. Without added cations both G3T and (G3T)_2_ demonstrate equilibrium transitions around the same temperature, ~30 °C (no cations, Fig. 7).

**Figure 8:**
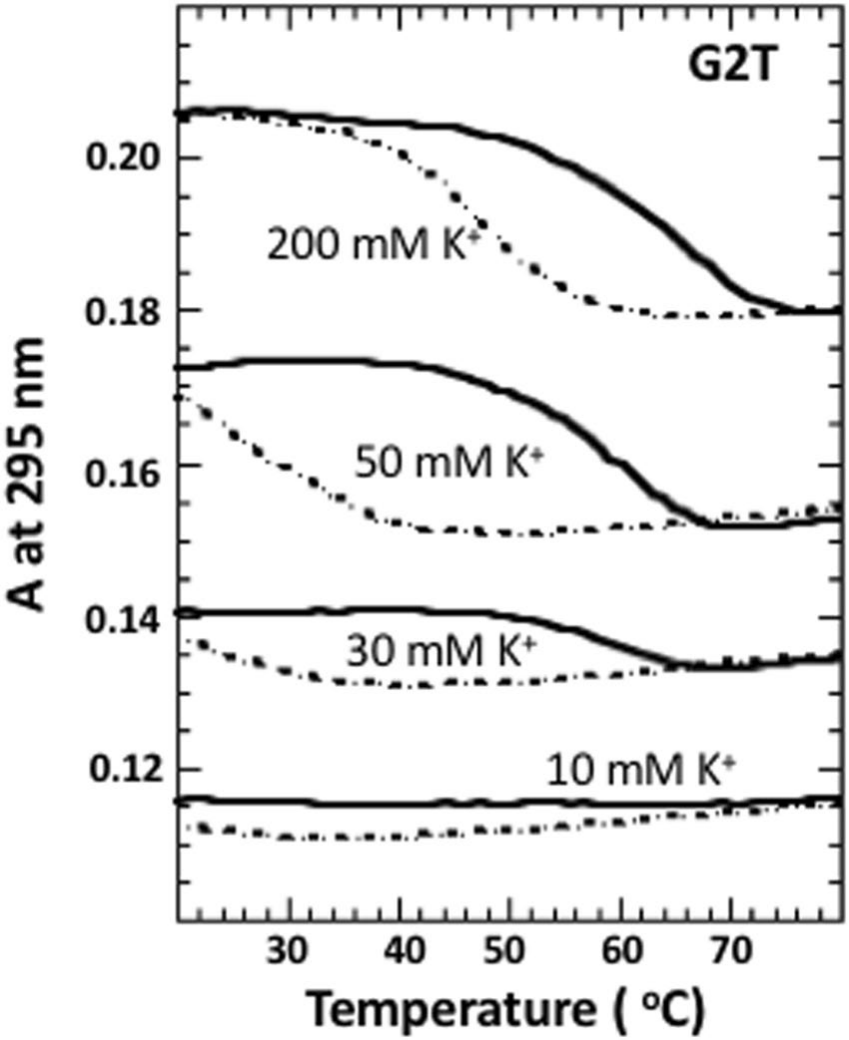
UV unfolding (solid) and refolding (dashed) curves of G2T in the presence of different amounts of KCl.

G2T domain didn’t show any detectable transition even in the presence of 10 mM K^+^ (Fig. 8). Melting experiments above 30 mM K^+^ demonstrate some transitions; increase in K^+^ concentration is accompanied by the increase in magnitude of the transition, however, thermal stability is independent on K^+^ concentration - all curves demonstrate transitions at ∼60 °C (Fig. 8). All cooling curves show hysteresis that decreases with increase of K^+^ concentration. G3T dimer, (G2T)_2_, demonstrates equilibrium transitions in the presence of 10 mM and 50 mM K^+^ ions with *T*_m_s of ∼65 °C and ∼75 °C (Fig. S3).

The quadruplex domains based on four G-tetrads, G4T2 and G4T3, in the presence of K^+^ ions, show higher stabilities in comparison with G2T, however demonstrate hysteresis indicating on polymorphic nature of the quadruplexes (Figs S4 and S5). The melting experiments of the dimers, (G4T2)_2_ and (G4T3)_2_, demonstrate hysteresis without any increase in stability (Figs S4 and S5).

## Discussion

### Quadruplex domain with three G-tetrads

As discussed above, the loop elongation in G3T domain is accompanied by strong destabilization and ruining of the uninterrupted nature of tmDNA. Therefore, in this section we investigate only G3T domain and test whether it is able to keep tmDNA architecture in the presence of different cations. In the following paragraph, we analyze already reported thermodynamic parameters on K^+^-G3T and K^+^-(G3T)_2_ and define experimental indicators of tmDNA architecture.

#### K^+^ ion

Earlier study of G3T and (G3T)_2_ revealed that K^+^ ions form so stable quadrulexes that unfolding experiments are limited to only less 0.5 mM cation concentration^4^. For instance, at 0.1 mM KCl and 0.5 °C/min heating rate, *T*_m_ of G3T is 55 °C, while (G3T)_2_ unfolds at ∼80 °C and refolds at ∼55 °C resulting in ∼25 °C hysteresis. The hysteretic loop disappears only at heating/cooling rate less than 0.02 °C/min. However, equilibrium *T*_m_ of (G3T)_2_ is still 15 °C higher than *T*_m_ of G3T^4^. Thus, dimerization of G3T is accompanied by strong thermal stabilization. CD profiles of G3T and (G3T)_2_ were exactly same corresponding to a typical all-parallel G-quartets with exclusively *anti* glycosyl bonds^17,21^ formed by chain-reversal T-loops^3,22^. Noteworthy feature of the CD spectra is that the molar CD amplitudes of (G3T)_2_, calculated per G3T unit, is increased by 2.6-fold from the level of G3T instead of expected 2-fold^1^. The extra CD signal is attributed to 5 stacking interaction in (G3T)_2_ *vs* 4 in two separated G3T^1^. Thus, based on these studies we have derived four experimental indicators of tmDNA formation: (i) fully reversible two-state transition of a quadruplex domain (i.e., G3T) at relatively low cation concentration (<1 mM); (ii) a significant increase in stability (~15 °C) upon the domain dimerization; (iii) exclusively all-parallel topology for both domains and dimers; and (iv) an extra CD signal due to dimerization, or more than 2-fold increase upon dimerization.

#### Na^+^ ion

While for K^+^-(G3T)_2_ the highest measurable cation concentration is 0.5 mM^4^, for Na^+^-(G3T)_2_ entire melting curve can be recorded in the presence of significantly higher concentrations (Fig. 5). At [Na^+^] <1 mM, we observe the hysteresis typical for K^+^-(G3T)_2_, while at 75 mM Na^+^ both G3T and (G3T)_2_ demonstrate equilibrium transitions at 65 °C and 85 °C, respectively (Fig. 5). Thus, the hysteretic behavior of (G3T)_2_ is due to low concentration of the quadruplex-forming cations. The equilibrium transition of (G3T)_2_ at 75 mM Na^+^ should be attributed to its non-specific binding to phosphate groups in addition to chelation between G-tetrads. The former neutralizes negative charge of the oligomer, decreases repulsion between G_3_-segments and facilitates quadruplex formation (see following paragraph for additional proves). CD profiles of Na^+^-G3T and Na^+^-(G3T)_2_ are identical to the profiles measured with K^+^ ions and demonstrate significant increase in CD amplitude upon dimerization (Fig. 3). Thus, Na^+^-G3T completely complies to all requirements for formation of tmDNA. Specifically, (i) Na^+^-G3T demonstrates two-state transitions at 0.1 mM cation concentration; (ii) dimerization is accompanied by ~20 °C stabilization; (iii) CD demonstrates only all-parallel topology for both G3T and (G3T)_2_; and (iv) almost 3-fold increased CD amplitude at 265 nm upon dimerization.

#### Mixture of K^+^ and Cs^+^ ions

The preceding sections suggest that the large hysteresis of K^+^-(G3T)_2_ is due to inability of the cation, at low concentrations, to neutralize negative charge of oligonucleotides and overcome repulsion between G_3_-segments upon the quadruplex formation. If our hypothesis is true, hysteresis of K^+^-(G3T)_2_ should disappear in the presence of CsCl, which is too large to enter the inner core of the quadruplexes, however can neutralize negative charge of the phosphates through surface binding. The melting experiments clearly indicates that the hypothesis is correct: in the presence of 1 mM KCl and 400 mM CsCl, the dimer demonstrates equilibrium transition with ~20 °C higher *T*_m_ than that of G3T (Fig. 6). Earlier Arrhenius analysis, performed for (G3T)_2_ in the presence of 0.1 mM K^+^ ions, suggested a highly specific two-state transition in which the folding and unfolding of the first G3T monomer is rate-limiting for both annealing and melting processes^4^. We suggest that the higher ionic strength accelerates simultaneous folding of both G3T domains probably initiated at the middle G6-segment.

#### Other monovalent cations

NH_4_^+^ ion with 143 pm radius is a favorable cation for quadruplex formation^7^. As expected, unfolding of G3T and (G3T)_2_ are similar to that of K^+^ and Na^+^: strong stabilization due to dimerization and large hysteresis (Fig. S6). CD profiles also fully comply to the requirements of tmDNA (Fig. 3). Thus, in the presence of NH_4_^+^ ions G3T forms tmDNA.

Adding 50 mM Li^+^ and Cs^+^ ions didn’t reveal any influence on the melting behavior and CD profiles of the oligonucleotides dissolved in just 10 mM Tris buffer (data not shown). This well agrees with the notion that radii of Li^+^ and Cs^+^ ions are too small and too large, respectively, to form G-tetrads.

#### Divalent cations

All divalent cations, including Mg^2+^ ions, demonstrate qualitatively similar results and suggest formation of tmDNA. Specifically, G3T dimerization is accompanied by increase in CD signal (Fig. 3) and strong thermal stabilization (Fig. 7). The quadruplex-folding ability of Mg^2+^ ion is unexpected since its ionic radius is too small. This observation is supported by the fact that (G3T)_2_ quadruplex can be folded even without any added cations. Specifically, CD profile of (G3T)_2_ dissolved in 10 mM Tris buffer at 20 °C is identical to the profile of K^+^-(G3T)_2_ at 50 °C (Fig. 3) and melting experiment demonstrates an equilibrium transition at ~30 °C (Fig. 7). Thus, it is quite possible that Mg^2+^ ions stabilize the quadruplex through non-specific binding at quadruplex surface without chelation between G-tetrads. Another argument, supporting this idea, comes from the fact that only Mg^2+^-(G3T)_2_ demonstrates hysteresis indicating on the weak stabilization of Mg^2+^ ions (Fig. 7); other divalent cations, capable of chelation (Ca^2+^, Sr^2+^ and Ba^2+^), show equilibrium transitions without any hysteresis. Interestingly, Mg^2+^-induced stabilization effect completely disappears upon adding CsCl (middle curves in Mg^2+^ panel of Fig. 7), characteristic to only non-specific interactions^23^. In contrast, adding of CsCl does not affect unfolding pattern of K^+^-G3T or K^+^-(G3T)_2_ (Fig. 6) since K^+^ binding is specific. As a final test, we performed additional experiments of (G3T)_2_ in the presence of Hexamminecobalt(III), Co(NH_3_)_6_^3+^. Chelation of this coordination compound, which represents six ammonia molecules covalently attached to the cobalt atom, can be completely excluded. As expected, influence of Co(NH_3_)_6_^3+^ ions on melting and CD profiles of G3T resembles the effects of Mg^+^ ions (Fig. S7).

The main conclusion of this section is that G3T-tmDNA demonstrates strong structural conservatism in the presence of all alkali and alkaline-earth cations. The tmDNA formation is so favorable that it forms at ambient temperatures without any added cations and can be stabilized only by non-specific interaction at the surface of tmDNA without specific chelation between G-tetrads.

### Quadruplex domain with two G-tetrads

Since single T-loops perfectly span over three G-tetrads, G2T domain represents the best candidate for tmDNA formation. UV melting experiments demonstrate non-equilibrium transitions even at [K^+^] = 200 mM (Fig. 8). The structure is so unstable that transitions aren’t detectable below 30 mM K^+^ (for comparison, at this concentrations G3T melts above 100 °C) (Fig. 8). In addition, increase in [K^+^] doesn’t shift the transitions to higher temperatures; all transitions take place at ~60 °C. It is clear that G2T forms very unstable and probably a polymorphic structure. Polymorphic nature of G2T is further supported by CD spectra, which suggest both parallel and anti-parallel topologies (Fig. 4). Interestingly, earlier study of a similar sequence (two G2T linked by TGTT) demonstrated at least eight monomeric quadruplex species that interconvert very slowly at room temperature^24^. UV melting of (G2T)_2_ shows relatively stable equilibrium transitions (Fig. S3), however, CD spectrum demonstrates polymorphic nature of the quadruplexes (Fig. 4). In the presence of Na^+^ ions, none of the sequences, G2T or (G2T)_2_, demonstrate formation of a stable quadruplexes even at 20 °C (Fig. 4).

G2T2 was ruled out from the list of candidates since it forms very unstable and only partially folded antiparallel quadruplex in the presence of K^+^ ions^11^.

Thus, quadruplex domains based on two G-tetrad are incapable of forming tmDNA.

### Quadruplex domain with four G-tetrads

The sequences containing G_4_ segments are well studied because they are part of telomeres: G4T2 (G_4_T_2_G_4_T_2_G_4_T_2_G_4_) in *Tetrahymena* and G4T4 (G_4_T_4_G_4_T_4_G_4_T_4_G_4_) in *Oxytricha*. In short, none of those studies demonstrate formation of parallel quadruplex with four G-tetrads. However, we still performed some experiments to test whether the dimers (i.e., (G4T2)_2_) are favoring tmDNA formation. In this section, we consider G_4_-tracks separated by T_1–4_ loops.

#### G4T

Earlier thermodynamic, optical and gel-mobility studies revealed that G4T forms a quadruplex with three G-tetrads connected by GT-loops and measured parameters are similar to that of G3T2^12^. Thus, G4T represents destabilized version of G3T.

#### G4T2

NMR structure of Na^+^-G4T2 reveals an antiparallel quadruplex containing only three G-tetrads connected by two lateral (GTTG and TTG) loops and a chain-reversal TT-loop^25^. Thermodynamic study of G4T2, performed for both Na^+^ and K^+^ ions, also corresponds to antiparallel quadruplex with only three G-tetrads^10^. Recent study of K^+^-G4C2 reveals formation of an antiparallel quadruplex with four G-tetrads connected by three lateral CC loops^26^. Interestingly, RNA analog of G4C2 forms all-parallel quadruplex, however with only three G-tetrads connected by GCC-loops^26^. The parallel fold of the RNA quadruplex is due to its structural constrains in adopting *syn* glycosyl bonds, which is required for antiparallel topology. Thus, even when a quadruplex domain is forced to adopt a parallel topology, and has sufficiently long loops, it is not able to incorporate more than three G-tetrads. These reported data are in general agreement with our experiments, which show no sign of formation of a homogenous all-parallel topology with four G-tetrads in G4T2 or (G4T2)_2_ (Fig. S4). Specifically, (i) unfolding experiments of G4T2 revealed that the transitions are non-sigmoidal with hysteresis indicating on more than two quadruplex species; (ii) CD profile corresponds mainly antiparallel topology (Fig. 4); (iii) experiments performed on (G4T2)_2_ demonstrate similar behavior without any increase in stability or changing topology from antiparallel to parallel (Figs 4 and S4). Thus, G4T2 is not able to form tmDNA.

#### G4T3

Our melting and CD experiments on G4T3 are similar to that of G4T2: non-equilibrium transitions of quadruplexes (Fig. S5) with antiparallel topology for both monomer and dimer (Fig. 4), suggesting incapability of G4T3 to form tmDNA.

#### G4T4

Two independent NMR studies of G4T4 demonstrates antiparallel topology with four G-tetrads^25,27^. We did not study (G4T4)_2_ sequence because studies performed on (G4T2)_2_ and (G4T3)_2_ clearly indicate that inability of all-parallel quadruplexes to incorporate more than three G-tetrads is not related with the loop length. Probably, sugar-phosphate backbone is not capable of vertical connection between top and bottom G-quartets (Fig. 2C).

Thus, quadruplex domains based on both two and four G-tetrads are incapable of tmDNA formation.

### PolyG is capable of tmDNA formation using G3G pattern

Role of nucleotides in the loop position of G3T was studied earlier, which demonstrated that substitution of C for T (C → T) does not have a measurable effect on thermal stability of the quadruplex, while purine substitutions A → T and G → T destabilize the quadruple by ~8 °C and ~5 °C per substitution^12,28–30^. The destabilization effect of purines can be explained by stronger stacking interactions with the adjacent Gs, which has to be overcome during rearrangement of unstructured oligonucleotide into the quadruplex. In agreement with this explanation, abasic nucleotides in loop positions show even a stabilization effect^12,30^. The destabilization effect of purines does not depend on the loop position and is additive (for instance, all three G → T substitutions destabilize G3T by ~15 °C^12,28–30^). None of the substitutions have any effect on the folding topology of G3T^12,28–30^. Thus, G3T tolerates any nucleotide substitution in the loop positions, and G3N, including G_15_ or G3G are capable of tmDNA formation. Since production of G_30_ is impractical through a chemical synthesis, we tested all purine oligonucleotide (G3A)_2_. As expected, (G3A)_2_ completely comply with all requirements of G3A-tmDNA (data not shown). Based on the facts that (i) G3A, which is ~10 °C less table than G3G, forms tmDNA and (ii) CD profiles of G3A and G3G are identical, we deduce that polyG, should be capable of G3G-tmDNA formation.

Several experimental evidences, that polydG folds into G3G-tmDNA, are detected in the earlier study of polyG.polyC duplex before and after strand-separation^31^: (i) AFM imaging clearly demonstrated that separated polyG strand folds into a monomolecular, continuous quadruplex (or G-wire) with all Gs involved in G-tetrads. The authors concluded formation of long antiparallel quadruplex with three short lateral loops^31^. Formation of such structure is highly unlikely, since it requires formation of three short loops at precisely defined positions within the same polyG strand. For instance, in ~5,000-nt long polyG, loops had to be created only at positions ~1,250, ~2,500 and ~3,750; (ii) statistical morphology analysis, performed on AFM images of polyG.polyC duplexes and G-wires prepared from the same starting material, demonstrates a 5-fold reduction in the length instead of the expected 4-fold reduction^31^. The discrepancy is perfectly explained by tmDNA in which exactly 1/5 of the nucleotides (3 out of 15) are utilized in loops; (iii) the reported CD spectrum is characteristic to all-parallel homogeneous quadruplex without any antiparallel topology^31^; (iv) in the presence or absence of cations G-wires showed same CD and UV profiles^31^, which is characteristic only to tmDNA (see Unique Properties of tmDNA: 1); (v) boiling of G-wires for one hour does not affect their gel-mobility even upon very fast annealing (tens of seconds)^31^, which is again characteristic only to tmDNA (see Unique Properties of tmDNA: 2).

### Unique properties of tmDNA

#### Unusually high stability and structural conservatism

In the presence of 1 mM K^+^ ions, G3T domain forms homogenous, all-parallel quadruplex and demonstrates equilibrium transition with *T*_m_ of 75 °C. Under the same experimental conditions, its dimer and higher order multimers unfold around the boiling temperature. The all-parallel topology of tmDNA (Fig. 1) is maintained in the presence of all alkali and alkaline-earth cations and even in the absence of added cations at ambient temperature. This kind of stability and structural conservatism is unprecedented in whole NA world.

#### No misfolding during rapid cooling

G_15_, (G3T)_2_ or (G_3_T)_7_ GGG do not show any misfolding after rapid cooling on ice (Fig. S2)^4^. The latter construct is a variant of (G3T)_2_ with T-insertion between G3T domains, G3T-T-G3T. Since is contains eight identical G_3_-segments, it is more inclined to misfold. For instance, it might form only one G3T domain with unstructured tales, i.e., G_3_TG_3_T-(G3T)-TG_3_TG_3_, or use G_3_T-segments as loops. However, it forms two perfectly folded G3T quadruplexes even upon rapid annealing on ice (Fig. S2). Structural reversibility upon rapid annealing is uncommon for nucleic acids, which require careful annealing to restore initial structures.

#### Highly programmable and predictable secondary structure

tmDNA structure formed from sequences with A, T or C loops can be programmed and predicted with 100% accuracy. In the case of polyG, the structure can be predicted by 100% accuracy if polyG length equals to *n* × 15 (i.e., G_30_, G_45_). Otherwise, one can predict number of G3G domains in tmDNA, however without exact positioning the ends of the structure. For instance, G_32_ might fold in GG-(G3T)_2_, G-(G3T)_2_-G or (G3T)_2_-GG. This kind of secondary structure predictability is unusual for biopolymers. For instance, only short DNA duplexes with specifically designed W-C base pairs can be predicted with 100% accuracy, while the highest accuracy for proteins is around 80%^32^.

#### Structure with exposed bases

tmDNA represents the first nucleic acid architecture, which has strictly defined building pattern with fully exposed nucleic bases similar to Pauling model of DNA^33^.

### Significance of tmDNA

#### Biology

The polyG tracts are common feature for many genomes. For instance, chromosome 2 of human genome contains 427-lt long sequence with 405 Gs^34^. Interestingly, in some genomes, i.e., *C*. *elegans* or *C*. *briggsae*, polyG segments are over-represented and distributed in a non-random pattern^35^. At all this positions G3G-tmDNA can be formed.

#### NA nanotechnologies

Quadruplexes show promise as a potential tool for nanoscale assembly^31,36–39^. However, programmed design of multimolecular quadruplexes is a significant challenge. Specifically, since the G-quartets are formed by guanines only, it is problematic to prevent slippage of the strands relative to each other similarly to a DNA duplex made of homopolymers. As a result, annealed product is a complex mixture of stacks of G-quartets of different lengths^31,38,39^. Since tmDNA is the monomolecular architecture (i) it is easily programmable, (ii) demonstrates favorable thermodynamics and kinetics, and (iii) avoids misfolding. As a result, tmDNA has strong potential in nanotechnologies that was already demonstrated in designing DNA nanoswitches^4^, high affinity coupling^5^ and driving force for endergonic reactions^6,40^.

#### Possible role during abiogenesis

One of the most striking aspect of the current work is that a non-specific homopolymer, polyG, can form a sophisticated secondary structure with a strictly defined building pattern. For instance, while G3T-tmDNA requires incorporation of thymidines at specific positions (Fig. 1) (achievable only by a programmable synthesis, i.e., enzymatic synthesis with a specific template), G3G-tmDNA can be produced by a simple condensation polymerization of guanosines (i.e., step-growth polymerization during abiogenesis). Interestingly, GGG codon codes the simplest amino acid glycine and interaction between them is considered as a starting point of the genetic code^41^. Emerging of the genetic code around polyG is supported by capability of guanines (free monomers) to form G-tetrads that are stacked on each other in a helical manner^42^. As a result, 5′ phosphate and 3′ hydroxyl termini of guanines of adjacent G-tetrads are juxtaposed, which facilitates non-enzymatic formation of phosphodiester bonds between them and transforms G-tetrad stacks of free guanines into a quadruplex with four oligoG strands. After dissociation of the quadruplex (i.e., by a temperature cycle, which is essential for the molecular evolution^43^), the short oligoG strands would spontaneously form long polymeric quadruplex (i.e., G-wire^38,44–46^), which would juxtapose 5′ phosphate and 3′ hydroxyl termini of the oligomers for ligation. Upon the following thermal cycles, long separated polyG would form monomolecular tmDNA and always refold into tmDNA upon later thermal cycles. At this point the abiogenic system would have a biopolymer (i) with strictly defined structural properties; (ii) resistant to harsh conditions; (iii) capable of refolding under almost any conditions; and (iv) characterized with a sophisticated surface (groves of different length, exposed bases) for interaction with other molecules (i.e., amino acids) or for self-assembly.

## Materials and Methods

DNA oligonucleotides were obtained from Integrated DNA Technologies. The concentration of the DNA oligonucleotides has determined by measuring UV absorption at 260 nm as described earlier^47^. All measurements were performed in 10 mM Tris-HCl, pH 8.7 with the ionic strength adjusted by addition of appropriate salts as indicated in the figure legends.

UV unfolding/folding experiments were recorded at 295 nm as a function of temperature using a Varian UV–visible spectrophotometer (Cary 100 Bio). CD spectra were obtained with a Jasco-815 spectropolarimeter. The devices were equipped with thermoelectrically-controlled cuvette holders. In a typical experiment, oligonucleotide stock solutions were mixed into the desired buffers in optical cuvettes. The solutions were incubated at 95 °C for a few minutes and annealed at room temperature for 2–3 min prior to ramping to the desired starting temperatures. Unless otherwise noted, UV melting experiments were performed at heating rate of 1 °C/min at 4 µM concentration of quadruplex domains (i.e., [G3T] = 4 µM and [(G3T)_2_] = 2 µM).

## Electronic supplementary material

Supplementary Information
