# Supplementary material for: Monomolecular tetrahelix of polyguanine with a strictly defined folding pattern
Source: Sci Rep. 2018 Jul 4;8:10115. doi: 10.1038/s41598-018-28572-x (PMC6031693; doi:10.1038/s41598-018-28572-x)
Supplement: Supplementary file 1 — Supplementary Information [file 41598_2018_28572_MOESM1_ESM.pdf]

SUPPORTING INFORMATION

Monomolecular tetrahelix of polyguanine with a strictly defined folding pattern

Besik Kankia

Department of Chemistry and Biochemistry, The Ohio State University, Columbus OH 43210, USA

* To whom correspondence should be addressed. Email: kankia.1@osu.edu, Telephone: 1-614-688-8799, Fax: 614-688-5402

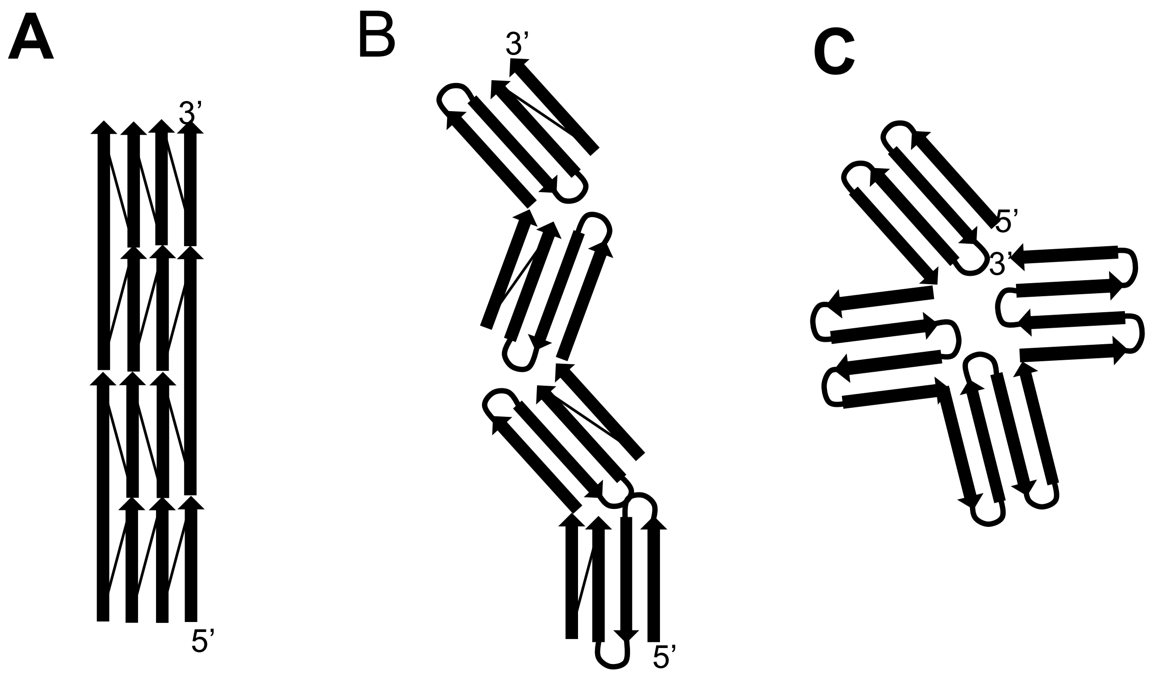

Figure S1. Models of all parallel tmDNA (**A**) and hypothetical structures with antiparallel quadruplex domains (**B** and **C**).

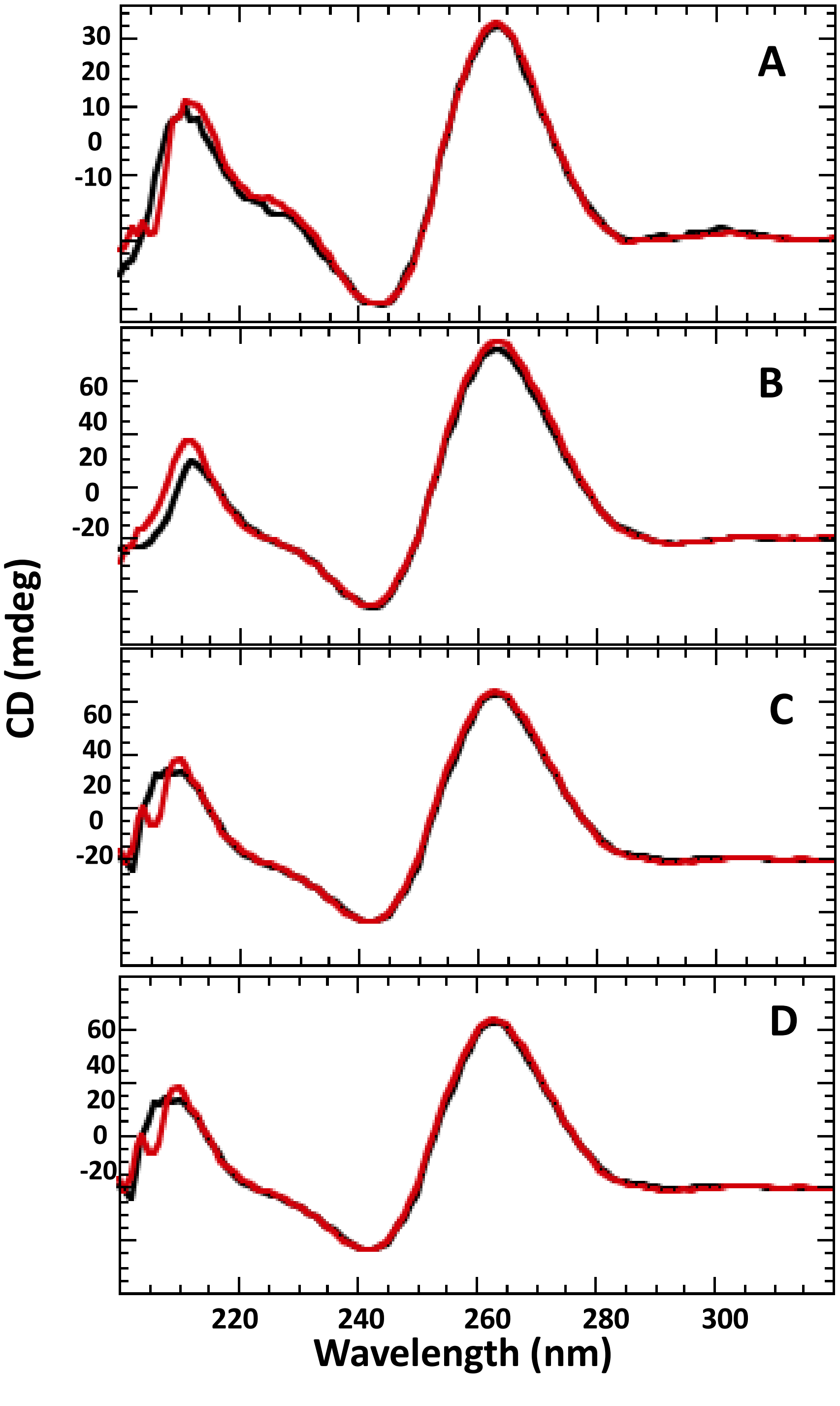

Figure S2. CD spectra of G_{15} (**A**), $(G3T)_2$ (**B**) and G3T-T-G3T (**C**) at 20 °C, in the presence of 50 mM KCl, prepared either by rapid cooling on ice (red) or slow annealing (black) demonstrating no misfolding during the rapid cooling. **Panel D** demonstrates CD spectra of $(G3T)_2$ in the absence of added cations prepared by rapid cooling on ice (red) and slow annealing (black). In all cases preparation does not have any influence on CD profiles. CD signals of G3T-T-G3T are exactly twice larger than that of G3T, demonstrating no misfolded structure with one G3T quadruplex with unstructured tales.

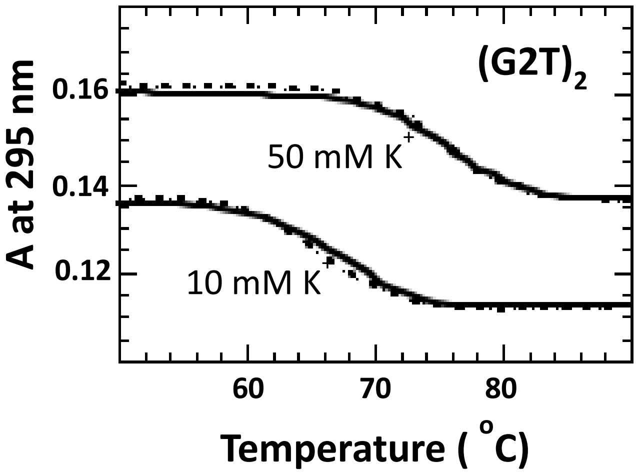

Figure S3. UV unfolding (solid) and refolding (dashed) curves of G2T in the presence of 10 mM and 50 mM KCl.

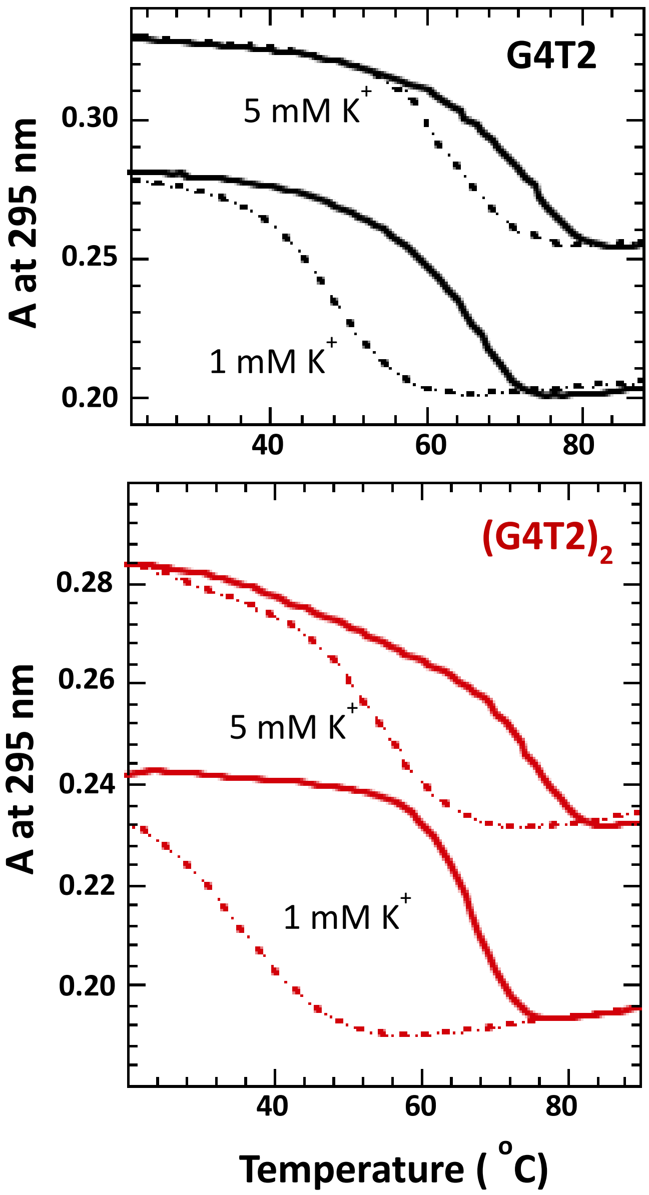

Figure S4. UV unfolding (solid) and refolding (dashed) curves of G4T2 (black) and $(G4T2)_2$ (red) in the presence of 1 mM and 5 mM KCl.

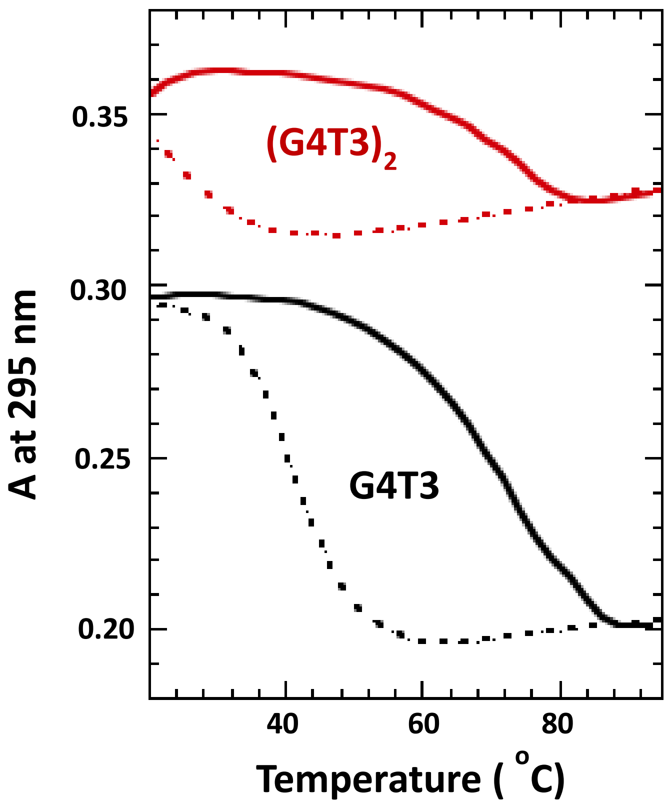

Figure S5. UV unfolding (solid) and refolding (dashed) curves of G4T3 (black) and $(G4T3)_2$ (red) in the presence of 1 mM KCl.

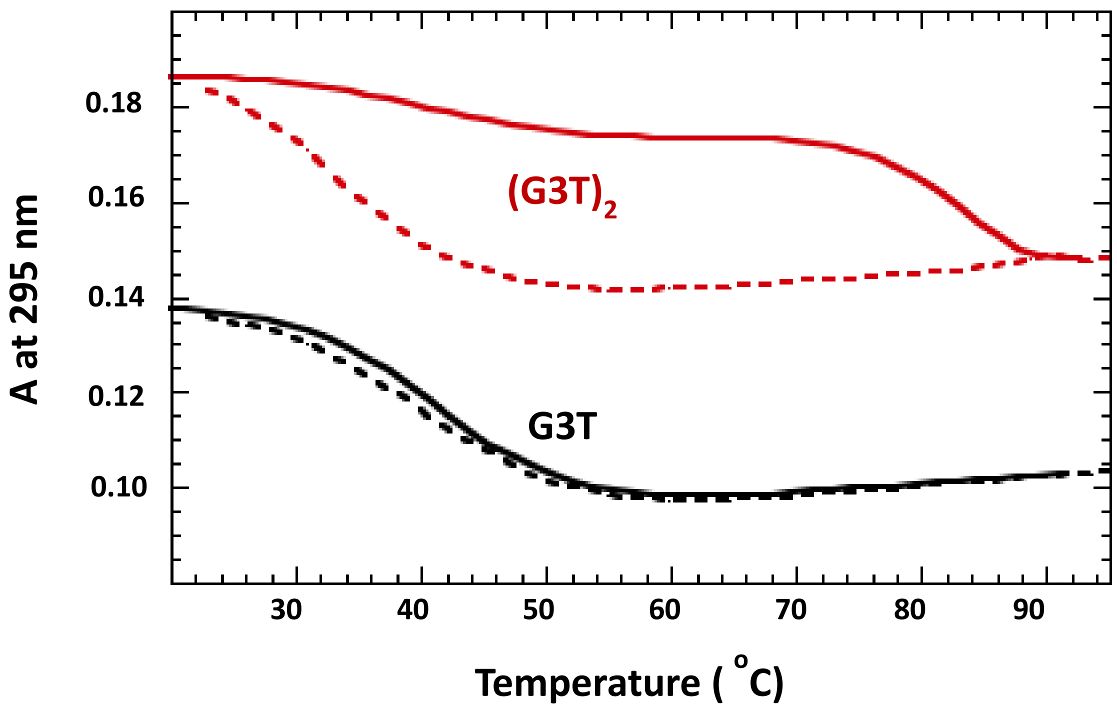

Figure S6. UV unfolding (solid) and refolding (dashed) curves of G3T (black) and $(G3T)_2$ (red) in the presence of 1 mM NH_4Cl.

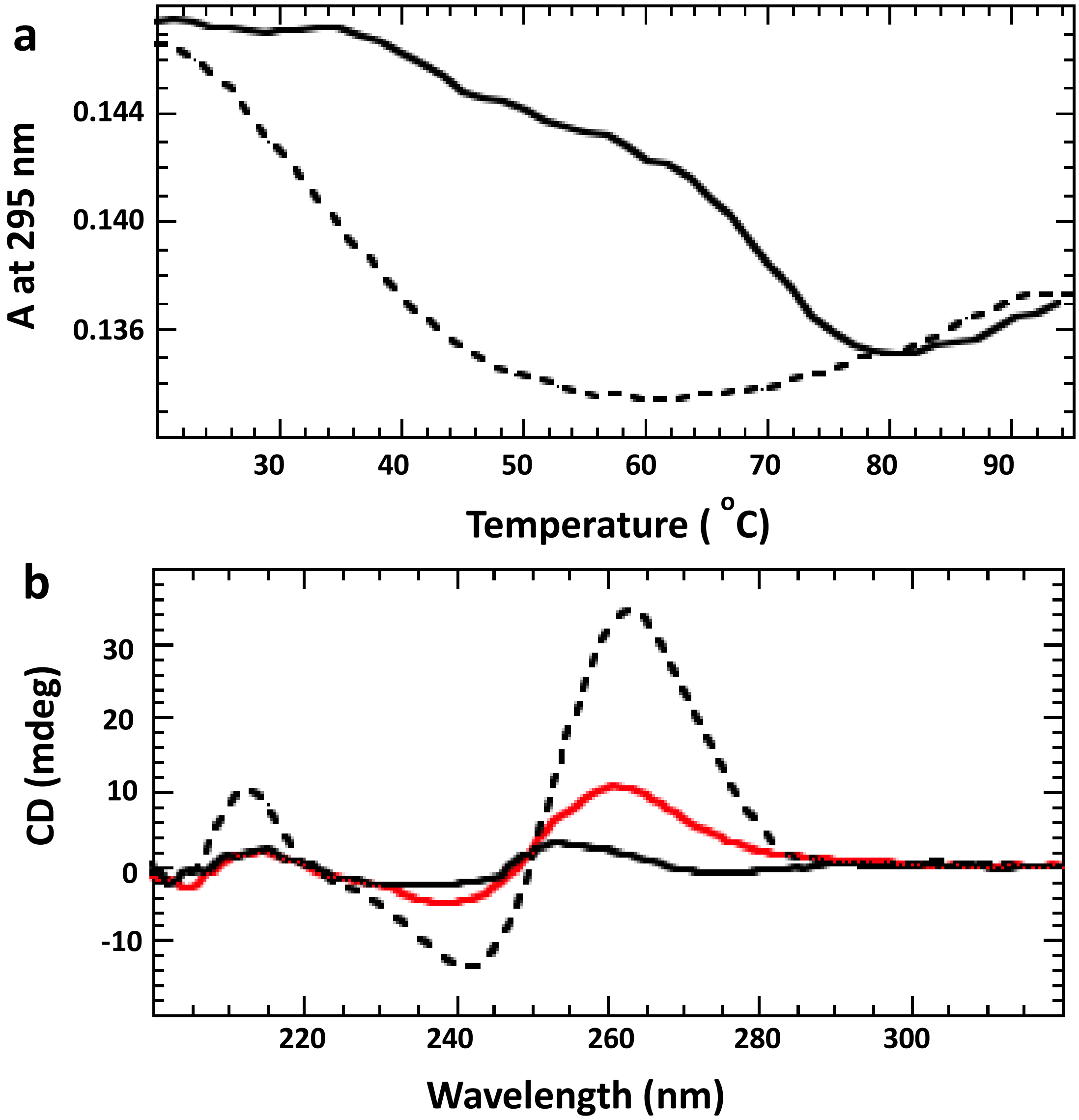

Figure S7. UV melting (**a**) and CD (**b**) profiles of $(G3T)_2$ of 2 µM in the presence of 5 µM $Co(NH_3)_6^{3+}$ ions. Measurements performed at 10 and 15 µM $Co(NH_3)_6^{3+}$ did not reveal reproducible data due to aggregation. It is known that trivalent $Co(NH_3)_6^{3+}$ induce DNA aggregation at very low concentrations (Kankia, Bloomfield 2001). UV melting demonstrates large hysteresis typical for K^+ and Mg^{2+} ions. Amplitude of the unfolding is roughly 3-fold less than the same parameter detected for K^+-$(G3T)_2$. Similarly, addition of $Co(NH_3)_6^{3+}$ is accompanied by moderate increase in CD amplitudes. Thus, these experiments demonstrate a stabilization of G3T-tmDNA by $Co(NH_3)_6^{3+}$ ions.
